# Grey and White Matter Correlates of Recent and Remote Autobiographical Memory Retrieval – Insights from the Dementias

**DOI:** 10.1371/journal.pone.0113081

**Published:** 2014-11-14

**Authors:** Muireann Irish, Michael Hornberger, Shadi El Wahsh, Bonnie Y. K. Lam, Suncica Lah, Laurie Miller, Sharpley Hsieh, John R. Hodges, Olivier Piguet

**Affiliations:** 1 School of Psychology, the University of New South Wales, Sydney, Australia; 2 Neuroscience Research Australia, Randwick, Sydney, Australia; 3 School of Medical Sciences, the University of New South Wales, Sydney, Australia; 4 Australian Research Council Centre of Excellence in Cognition and its Disorders, Sydney, Australia; 5 School of Psychology, the University of Sydney, Sydney, Australia; 6 Neuropsychology Unit, Royal Prince Alfred Hospital, and Central Clinical School, University of Sydney, Sydney, Australia; 7 Department of Clinical Neuroscience, University of Cambridge, Cambridge, United Kingdom; IIBB/CSIC/IDIBAPS, Spain

## Abstract

The capacity to remember self-referential past events relies on the integrity of a distributed neural network. Controversy exists, however, regarding the involvement of specific brain structures for the retrieval of recently experienced versus more distant events. Here, we explored how characteristic patterns of atrophy in neurodegenerative disorders differentially disrupt remote versus recent autobiographical memory. Eleven behavioural-variant frontotemporal dementia, 10 semantic dementia, 15 Alzheimer's disease patients and 14 healthy older Controls completed the Autobiographical Interview. All patient groups displayed significant remote memory impairments relative to Controls. Similarly, recent period retrieval was significantly compromised in behavioural-variant frontotemporal dementia and Alzheimer's disease, yet semantic dementia patients scored in line with Controls. Voxel-based morphometry and diffusion tensor imaging analyses, for all participants combined, were conducted to investigate grey and white matter correlates of remote and recent autobiographical memory retrieval. Neural correlates common to both recent and remote time periods were identified, including the hippocampus, medial prefrontal, and frontopolar cortices, and the forceps minor and left hippocampal portion of the cingulum bundle. Regions exclusively implicated in each time period were also identified. The integrity of the anterior temporal cortices was related to the retrieval of remote memories, whereas the posterior cingulate cortex emerged as a structure significantly associated with recent autobiographical memory retrieval. This study represents the first investigation of the grey and white matter correlates of remote and recent autobiographical memory retrieval in neurodegenerative disorders. Our findings demonstrate the importance of core brain structures, including the medial prefrontal cortex and hippocampus, irrespective of time period, and point towards the contribution of discrete regions in mediating successful retrieval of distant versus recently experienced events.

## Introduction

The ability to reminisce on events from the past represents a unique expression of the episodic memory system, and one that is essential for a sense of identity and continuity across subjective time [1]. Autobiographical memory (ABM) refers to the complex ability to retrieve personally experienced events from the past imbued with a sense of recollection and situated within a coherent spatiotemporal context [2], [3]. The recollection of ABMs relies upon the episodic memory system, permitting us to retrieve events from the past that are bound within a unique time and place, for example “My first holiday abroad with my family.” While self-referential in nature, ABMs also contain general conceptual knowledge or semantic memory, derived from the abstraction of content from experiences, for example “Paris is the capital of France.” ABMs therefore necessarily contain episodic and semantic elements [4], [5], as well as rich contextual sensory perceptual details and emotional salience, which facilitate the mental reliving of the original event [6]–[8].

Given the multifaceted nature of these memories, it is not surprising that a widespread neural network is implicated in the retrieval of autobiographical episodes from the past [9]. Functional neuroimaging studies in healthy individuals converge to reveal a distributed network of regions subtending successful ABM retrieval. Importantly, this core network includes medial temporal lobe (MTL) structures such as the hippocampus and surrounding parahippocampal cortices, lateral temporal lobe cortices, posterior parietal regions including the posterior cingulate cortex and precuneus, as well as frontal regions such as the medial prefrontal cortex (PFC) [9]–[11]. Crucially, these studies confirm that co-activation of multiple brain regions must occur to support successful ABM retrieval.

One outstanding issue in the literature concerns the extent to which specific brain regions within the ABM core network are differentially recruited during the retrieval of recent versus distant memories. This issue is of central relevance for elucidating how memories are consolidated over time. The standard consolidation theory [12] holds that the hippocampus plays a time-limited role in the storage and retrieval of ABMs, with memories becoming increasingly independent from the MTL and relying on neocortical areas following consolidation. In contrast, the multiple trace theory [13], [14] proposes that the hippocampus plays a permanent role in the retrieval of detailed and vivid episodic memories irrespective of remoteness of the memory. The evidence to date largely favours the multiple trace theory, with most neuroimaging studies demonstrating hippocampal activation during ABM retrieval from both recent and remote time periods [15]–[20].

While the hippocampus has tended to be the focus of most ABM neuroimaging studies, other brain regions within the ABM core network are likely to be sensitive to the age of the memories recalled [11]. For example, midline posterior regions, including the retrosplenial and posterior cingulate cortex, have consistently been shown to exhibit greater activation for retrieval of recent compared with remote ABMs [8], [15], [19], [21]. Several factors have been proposed to account for this preferential recruitment during recent recall, including the retrieval of self-referential information, generation of visual imagery, as well as increased emotional processing and recollection for more recent events [11]. Similarly, frontal cortical regions, including the medial PFC, have been found to preferentially activate with increasing recency of ABMs [10], [22] although this finding has not been consistently replicated [16], [21]. Finally, it has been suggested that, over time, a process of semanticisation occurs whereby episodic ABMs are transformed into less detailed, schematic memories more akin to semantic representations [23]. By this view, recent memories are more likely to encompass sensory-perceptual elements [24], whereas remote ABMs represent an abstracted or semanticised gist of the formerly evocative event [25]. Accordingly, these remote ABM schematic accounts are posited to draw heavily upon regions specialised for semantic processing in the brain [4]. Differential involvement of the lateral temporal cortices for remote memories, however, has not been consistently reported [3], [9], [19]. Thus, while functional neuroimaging studies have clarified the overall neuroanatomy of the core network required to support ABM retrieval, it remains unclear which components of this network are differentially involved in recent versus remote retrieval.

One approach to identify the key structures required for recent versus remote memory retrieval, is to study the disruption of ABM in neurodegenerative disorders [26]. It is well established that ABM is severely compromised in Alzheimer's disease (AD) with temporal gradients typically observed whereby remote memories are recalled in significantly better detail compared with more recent time periods [7], [27], [28] although flat profiles have also been reported [29], [30]. Studies incorporating neuroimaging analyses have pointed to the pivotal role of hippocampal and surrounding medial temporal lobe degeneration in the origin of ABM dysfunction in AD [31]–[33]. In contrast, the syndrome of semantic dementia (SD) is associated with the converse profile of ABM retrieval, whereby retrieval of recent events is typically disproportionally better compared with distant epochs [28], [29], [34] although flat profiles have also been noted [35], [36]. The loss of remote memories in SD has been ascribed to the progressive deterioration of the lateral temporal cortices, disrupting semantic information that comprises, or is required to access, the memory trace [4], [37]. Finally, in the behavioural variant of frontotemporal dementia (bvFTD), the majority of studies have revealed a flat profile, indicating global deficits in ABM irrespective of time period [28], [29], [38]. These impairments are attributable predominantly to medial prefrontal and lateral temporal dysfunction [39], with recent evidence pointing towards significant medial temporal lobe involvement in the genesis of episodic memory impairments in bvFTD [40].

The objective of the present study was to explicate the neural substrates of recent and remote ABM retrieval on the Autobiographical Interview (AI) [41] using the disease syndromes of AD, SD, and bvFTD as lesion models for this process. By incorporating structural neuroimaging analyses, we sought to clarify how changes in grey matter density, assessed using voxel-based morphometry, and alterations in white matter connectivity, as indicated by fractional anisotropy values extracted from diffusion tensor imaging, differentially associate with the retrieval of recent versus remote ABM retrieval. Based on previous studies, we predicted frontal and medial temporal lobe involvement irrespective of epoch. Integrity of the lateral temporal cortices was predicted to be strongly associated with remote ABM retrieval, suggestive of a crucial role for semantic processing in the retrieval of old memories. In contrast, we expected that integrity of midline posterior parietal structures, important for self-referential processing and visual imagery, would correlate with the retrieval of recent events.

## Methods

### Participants

Thirty-six dementia patients (bvFTD = 11; SD = 10; AD = 15) and 14 education-matched healthy controls were recruited through FRONTIER at Neuroscience Research Australia, Sydney. All dementia patients met the relevant clinical diagnostic criteria for bvFTD [42], SD (also known as semantic variant Primary Progressive Aphasia) [43] or AD [44]. Clinical diagnoses were established by multidisciplinary consensus among a senior neurologist, clinical neuropsychologist, and occupational therapist based on extensive clinical investigations, cognitive assessment, report of activities of daily living, and structural neuroimaging. Briefly, bvFTD patients presented with decline in behaviour and interpersonal functioning accompanied by loss of insight, increased apathy, and emotional blunting. To exclude potential phenocopy cases in the bvFTD group [45], only those cases showing evidence of progression over time, as reported by their caregivers, and with atrophy on structural MRI scans, were included. SD patients exhibited progressive loss of word meaning with significant naming and comprehension impairments, as well as prosopagnosia and/or associative agnosia, with relatively intact everyday memory. Finally, AD patients displayed significant episodic memory loss, in the context of preserved personality and behaviour.

Healthy controls were recruited from the FRONTIER research volunteer panel and local community clubs. All controls scored 0 on the Clinical Dementia Rating scale (CDR) [46], and 88 or above on the Addenbrooke's Cognitive Examination-Revised (ACE-R) [47].

Exclusion criteria for all participants included prior history of mental illness, significant head injury, movement disorders, cerebrovascular disease, alcohol and other drug abuse, and limited English proficiency.

### Ethics Statement

This study was conducted in accordance with the Declaration of Helsinki. Ethical approval was obtained from the Human Research Ethics Committee of the South Eastern Sydney and Illawarra Area Health Service (HREC 10/126) and the University of New South Wales Human Research Ethics Advisory panel D (Biomedical, ref. # 10035). All participants, or their person responsible, provided written informed consent. Capacity to provide informed consent was established by asking participants to signify that they understood the purpose of the research visit by explaining the proposed research in their own words. In the event that patients lacked the capacity to provide informed consent, written informed consent was obtained from the patient's next of kin or legally authorised representative. Withdrawal from the study was permitted at any time if either the patient or the family member elected to discontinue. Participants volunteered their time and were reimbursed for travel costs.

### Behavioural testing

#### General cognitive screening

Participants were assessed across the following neuropsychological tests: ACE-R as a general measure of global cognitive functioning, delayed recall on the Rey Auditory Verbal Learning Test (RAVLT) [48] to measure verbal episodic recall; delayed recall of the Rey Complex Figure (RCF) [49] as an index of non-verbal episodic memory, and the Trail Making Test (Parts B-A) [50] as an index of executive function. Verbal semantic processing was assessed using verbal letter fluency (F, A, S) [51], and the Naming and Comprehension subtests from the Sydney Language Battery (SydBat) [52]. The functional status of patients was determined using the Frontotemporal Dementia Functional Rating Scale (FRS) [53], which is a dementia staging tool sensitive to changes in functional abilities and presence of neuropsychiatric symptomatology.

### Assessment of Autobiographical Memory

The procedure for this study has been described in detail elsewhere [29]. Briefly, a shortened version of the Autobiographical Interview (AI) [41] was used to examine episodic autobiographical memory retrieval from across four life epochs; Teenage Years (11–17 years), Early Adulthood (18–35 years), Middle Adulthood (35–55 years), and Recent period (within the last year). The Early Childhood epoch (up to age 11) of the original AI test was omitted to shorten the overall test session and reduce the burden of testing on patient groups.

The AI was administered according to the standardised protocol. Briefly, participants were instructed to provide a detailed description of a personally experienced event that occurred at a specific time and place from each of the four life periods. In the case of participants who were unable to spontaneously retrieve a suitable event, a list of typical events for each time period was presented. In keeping with the original protocol, the level of retrieval structure was manipulated across three conditions; Free Recall, General Probe, and Specific Probe [41]. Participants first spoke extemporaneously about the event in question (free recall), following which general probes were used to encourage greater retrieval of detail. Finally, specific probes targeting five contextual details categories were provided (Event, Time, Place, Perceptual, Emotion/Thoughts). To avoid the contamination of subsequent memories, the specific probe condition was administered after all events had been retrieved via the free recall and general probe conditions. All interviews were digitally recorded for subsequent transcription and scoring.

#### Scoring of autobiographical memories

Following the original AI protocol, retrieved events were first segmented into informational bits or details, classified as a unique occurrence, observation or thought, typically expressed as a grammatical clause [41]. Each detail was then categorised as “internal” or “external”, representing episodic and semantic memory, respectively. Here, we focused on the retrieval of internal (i.e., episodic) details as an index of overall autobiographical event recollection Internal details were those details relating directly to the main episode, located within a specific spatiotemporal context, and reflected episodic re-experiencing [41]. We constrained our focus to the high retrieval support condition (Probed recall) to reduce the demands on generative search processes in the patient groups and to ensure that specific contextual details were elicited for each retrieved event.

To investigate potential differences across time periods, we averaged internal details retrieved across Teenage Years, Early Adulthood, and Middle Adulthood to create a Remote period composite score, which was then compared with Recent memory performance. The scores of interest in the present context were therefore: (i) Remote Total Retrieval and (ii) Recent Total Retrieval.

#### Statistical analyses

Cognitive data were analysed using IBM SPSS Statistics (Version 21.0). Multivariate analyses of variance (MANOVA) with Sidak post hoc tests were used to explore main effects of Group (Controls, bvFTD, SD, AD) for all general cognitive tests. The rationale for using the Sidak modification of the traditional Bonferroni post hoc test is that the statistical power of the analyses is not affected [54]. Performance on the Autobiographical Interview was analysed using one overall repeated measures MANCOVA, with age as a covariate, in which main effects of Epoch (Recent, Remote), and Group, as well as relevant interactions were explored. Chi-squared tests (*X^2^*), based on the frequency patterns of dichotomous variables (e.g., sex), were also used.

### Imaging acquisition

All participants underwent whole-brain T1- and diffusion- weighted images using a 3T Philips MRI scanner with standard quadrature head coil (8 channels).

The 3D T1-weighted images were acquired using the following sequences: coronal orientation, matrix 256×256, 200 slices, 1×1 mm^2^ in-plane resolution, slice thickness 1 mm, echo time/repetition time = 2.6/5.8 ms, flip angle α = 19°.

The diffusion-weighted sequences were acquired as follows: 32 gradient direction diffusion-weighted sequence (repetition time/echo time/inversion time: 8400/68/90 ms; *b*-value = 100 s/mm^2^; 55 2.5 mm horizontal slices, end resolution: 2.5×2.5×2.5 mm^3^; field of view 240×240 mm, 96×96 matrix; repeated twice). Two diffusion tensor imaging sequences were acquired for each participant, which were subsequently averaged. All scans were then visually inspected for field inhomogeneity distortions and corrected for eddy current distortions. Diffusion tensor models were fitted at each voxel via FMRIB's software library (http://fsl.fmrib.ox.ac.uk/fsl/fslwiki/FDT) which resulted in the creation of maps of three eigenvalues (λ1, λ2, λ3) allowing the calculation of fractional anisotropy for each participant.

### Voxel-based morphometry analysis

Three-dimensional T1-weighted sequences were analysed with FSL-VBM, a voxel-based morphometry analysis [55], [56] using the FSL-VBM toolbox from the FMRIB software package (http://fsl.fmrib.ox.ac.uk/fsl/fslwiki/FSLVBM/UserGuide) [57]. The VBM technique was used to identify grey matter density changes across groups on a voxel-by-voxel basis. Briefly, the brain extraction tool (BET) [58] was used to extract structural MR images, following which, tissue segmentation was carried out on the brain extracted images using FMRIB's Automatic Segmentation Tool (FAST) [59]. The FMRIB non-linear registration approach (FNIRT) [60], [61] was then used to align the resulting grey matter partial volumes to the Montreal Neurological Institute standard space (MNI152), using a b-spline representation of the registration warp field [62]. A study-specific template was created using the resulting images, to which the native grey matter images were re-registered nonlinearly. To correct for local expansion or contraction, the registered partial volume maps were then modulated by dividing by the Jacobian of the warp field. Finally, the modulated segmented images were smoothed with an isotropic Gaussian kernel with a sigma of 3 mm.

A voxel-wise general linear model was employed to investigate grey matter intensity differences via permutation-based non-parametric testing [63] with 5000 permutations per contrast. Differences in cortical grey matter intensities between patients (bvFTD, SD, and AD) and Controls were assessed using regression models with separate directional contrasts (i.e., t-tests). Clusters were extracted using the threshold-free cluster enhancement method and corrected for multiple comparisons using Family-Wise Error at *p*<.001. An overlap analysis was conducted to identify spatial overlap between regions of grey matter intensity commonly affected across the patient groups. The statistical maps generated from the atrophy analyses were scaled using a threshold of *p*<.001, following which, the scaled contrasts were multiplied to create an inclusive, or overlap, mask across groups.

Next, correlations between performance on the Autobiographical Interview and grey matter intensity were investigated using the “randomise” permutation-based inference tool in FSL for all participant images combined. This procedure was adopted to increase the study's statistical power to detect brain-behaviour relationships across the entire brain by achieving greater variance in behavioural scores [64], [65]. For additional statistical power, a covariate only general linear statistical model was employed in which group effects were not taken into account. Two separate GLMs were run; (i) Remote ABM performance with age included as a nuisance variable (1,0) and (ii) Recent ABM performance with age included as a nuisance variable (1,0). A positive t-contrast was used in the covariate model, providing an index of positive association between grey matter density and ABM scores. An unbiased voxel-wise whole-brain approach was used across all atrophy and covariate VBM analyses. Anatomical locations of significant results were overlaid on the MNI standard brain, with maximum coordinates provided in MNI stereotaxic space. Anatomical labels were determined with reference to the Harvard-Oxford probabilistic cortical atlas. For all covariate analyses, a threshold of 300 contiguous voxels was used, uncorrected at the *p*<.001 threshold. Similar to the atrophy analyses, an overlap analysis was conducted using the statistical maps generated from the recent and remote ABM contrasts to identify common grey matter regions implicated in recent and remote ABM retrieval. In addition, an exclusive masking procedure was employed to identify regions uniquely associated with retrieval in the recent and remote time periods. The scaled images were subsequently divided by each other to create an exclusive mask for each ABM time period.

### Diffusion tensor imaging analysis

Tract-based Spatial Statistics [66] from the FMRIB software library were used to perform a skeleton-based analysis of white matter fractional anisotropy. Fractional anisotropy maps for each participant were eddy current corrected and co-registered using non-linear registration (FNIRT) [60], [61] to the MNI standard space using the FMRIB58 fractional anisotropy template. Due to the coarse resolution of diffusion tensor imaging data (i.e., 2.5 mm^3^) the template was subsampled at 2 mm^3^. Following image registration, the fractional anisotropy maps were averaged to produce a group mean fractional anisotropy image. Then, a skeletonization algorithm [66] was applied to define a group template of the lines of maximum fractional anisotropy, corresponding to the centres of white matter tracts. Fractional anisotropy values for each participant were projected onto this group template skeleton, and extracted for use in subsequent correlation analyses with the ABM variables. Clusters were tested using permutation-based non-parametric testing as outlined for the voxel-based morphometry analyses. Age was included as a nuisance variable in these analyses. Clusters are reported using the threshold-free cluster enhancement method and corrected for Family-Wise Error at *p*<.05. Anatomical labels were determined with reference to the John Hopkins University white matter atlas and the ICBM-DTI-WM atlas labels integrated into FSLview [67], [68].

## Results

### Demographics

An overall group difference was evident for age (*F*(3, 50) = 4.410, *p* = .008), reflecting the fact that Controls were on average 9 years older than the bvFTD group (*p* = .011). The patient groups were matched for age (all *p* values >.05). All participant groups were matched for total years of education (*F*(3, 50) = .438, *p* = .727). A significant difference was evident for sex distribution (χ^2^ (3) = 9.326, *p* = .025), driven by the fact that significantly more females were present in the Control sample. Importantly, the patient groups were matched for disease duration (i.e., months elapsed since symptom onset, *p* = .109; AD versus bvFTD, *p* = .909; AD versus SD, *p* = .113; SD versus bvFTD, *p* = .381). The patients were further matched for functional status (FRS: *F*(2, 35) = 3.024, *p* = .063) although the suggestion of a greater functional impairment in bvFTD relative to SD (*p* = .059) was noted.

### General cognitive functioning

Neuropsychological testing revealed profiles characteristic of each patient group (Table 1). Briefly, all patient groups were significantly impaired on the ACE-R screening measure (*F*(3, 50) = 18.575, *p*<.0001) relative to Controls (bvFTD, *p* = .002; SD, *p*<.0001; AD, *p*<.0001). SD patients exhibited disproportionate deficits on the ACE-R in comparison with the bvFTD (*p* = .013) and AD (*p* = .056) groups, reflecting the verbal loading of this task. No differences were evident between the bvFTD and AD groups on the ACE-R (*p* = .968).

**Table 1:** Demographic and clinical characteristics of study cohort.

Demographics and cognitive tests	bvFTD (n = 11)	SD (n = 10)	AD (n = 15)	Controls (n = 14)	Group effect	Post hoc test
Sex (M/F)	9∶2	8∶2	12∶3	5∶9	*	
Age (years)	62.4 (7.3)	64.0 (8.9)	68.3 (8.8)	72.2 (3.9)	**	Controls>bvFTD
Education (years)	11.9 (3.0)	12.8 (3.0)	12.8 (2.9)	11.9 (2.4)	n/s	n/s
Disease duration (months)	44.2 (30.2)	61.8 (23.5)	37.6 (27.1)	n/a	n/s	n/s
FRS	−0.01 (1.5)	1.6 (1.4)	0.9 (1.6)	n/a	n/s	n/s
ACE-R (100)	76.5 (7.6)	61.3 (14.0)	73.2 (14.1)	93.4 (4.0)	***	Controls>PatientsbvFTD, AD>SD
RAVLT delayed recall (15)	2.1 (3.2)	n/a	2.2 (2.7)	10.7 (2.3)	***	Controls>AD, bvFTD
RCF 3 min recall (36)	6.9 (5.6)	13.8 (8.4)	3.4 (4.4)	16.5 (4.3)	***	Controls>AD, bvFTDSD = Controls
Naming (30)	20.0 (7.5)	5.6 (4.5)	21.0 (5.3)	25.6 (2.3)	***	Controls>SD
Comprehension (30)	23.7 (8.0)	21.9 (5.2)	25.7 (3.9)	28.7 (1.6)	*	Controls>SD
Letter Fluency	23.9 (12.3)	26.6 (12.3)	31.5 (14.5)	38.9 (10.9)	*	Controls>bvFTD
Trail Making Test Part B-A (s)	102.4 (68.8)	79.2 (81.2)	93.1 (57.1)	54.0 (32.1)	n/s	n/s

Maximum score for each test in brackets where applicable.

*p<.05;

**p<.005;

***p<.0001;

n/s = not significant; n/a = not applicable; bvFTD = behavioural-variant frontotemporal dementia; SD = semantic dementia; AD = Alzheimer's disease; FRS = Frontotemporal Dementia Functional Rating Scale; ACE-R = Addenbrooke's Cognitive Examination Revised; RAVLT = Rey Auditory Verbal Learning Test; RCF = Rey Complex Figure. FRS data not available for 1 AD patient; Trails data available for 9 AD, 10 bvFTD patients, and 13 Controls; Naming data available for 13 Controls; Comprehension data available for 9 bvFTD patients and 13 Controls; RAVLT not administered in SD patients due to the high verbal loading of the task; RCF recall data available for 10 bvFTD and 9 SD patients; Letter fluency data available for 14 AD patients.

Looking at each patient group separately, bvFTD patients showed significant impairments in verbal letter fluency (*p* = .030), delayed verbal and non-verbal episodic memory retrieval (RAVLT, *p*<.0001; RCF, *p* = .001) in the context of relatively spared Naming (*p* = .066) and Comprehension (*p* = .113) performance in comparison with Controls. No significant differences between bvFTD and Control participants were observed on the Trail Making Task (Part B-A) (*p* = .308).

The SD group was characterised by significant impairments in semantic Naming (*p*<.0001), and Comprehension (*p* = .014) with relatively preserved delayed non-verbal episodic memory (RCF, *p* = .850), executive functioning (*p* = .900), and letter fluency (*p* = .129) when compared with Controls.

Finally, AD patients demonstrated significant episodic memory impairments across verbal (RAVLT, *p*<.0001) and non-verbal (RCF, *p*<.0001) domains in the context of relatively preserved Naming (*p* = .132), Comprehension (*p* = .531), executive functioning (*p* = .586), and letter fluency (*p* = .556) in comparison with Controls.

### Autobiographical Memory Performance

**Figure 1:**
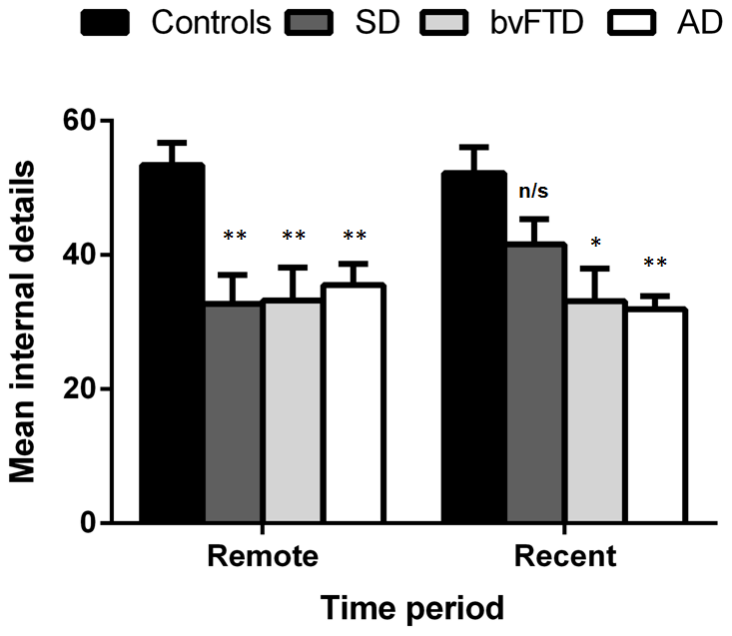
Total internal (episodic) details retrieved across remote and recent periods on the Autobiographical Interview. Error bars represent the standard error of the mean. * *p*<.05; ** *p* = .001; n/s = non-significant. Group differences refer to contrasts between patient groups and Controls. No significant differences were evident between the patient groups for remote or recent retrieval.

Figure 1 illustrates overall performance under high retrieval support (Probed Recall) across recent and remote time periods on the Autobiographical Interview for all participant groups. A repeated measures MANCOVA revealed that for overall internal details, a significant main effect of Group was evident (*F*(3, 45) = 8.794, *p*<.0001). This group effect reflected the fact that Controls recalled significantly more internal details than all patient groups irrespective of epoch, (AD, *p*<.0001; bvFTD, *p* = .001; SD, *p* = .007).

The repeated measures MANCOVA further revealed a significant Epoch×Group interaction (*F*(1, 45) = 3.746, *p* = .017). A main effect on the threshold of significance was found for Epoch (*F*(1, 45) = 3.858, *p* = .056), indicating that while all patients showed significant impairments for remote retrieval (AD, *p* = .001; bvFTD, *p* = .001; SD, *p* = .001), SD patients scored in line with Control performance for recent retrieval (SD, *p* = .279; AD, *p* = .001; bvFTD, *p* = .007). Further, within-group comparisons revealed that SD patients recalled significantly more internal details in the recent relative to remote periods (*p* = .005), while all other participant groups demonstrated equivalent ABM performance across time periods (AD, *p* = .155; bvFTD, *p* = .548; Control, *p* = .288). No significant differences were evident between patient groups for remote or recent retrieval (all *p* values >.4).

### Voxel-based morphometry analyses

#### Grey matter density profiles in patient groups

**Figure 2:**
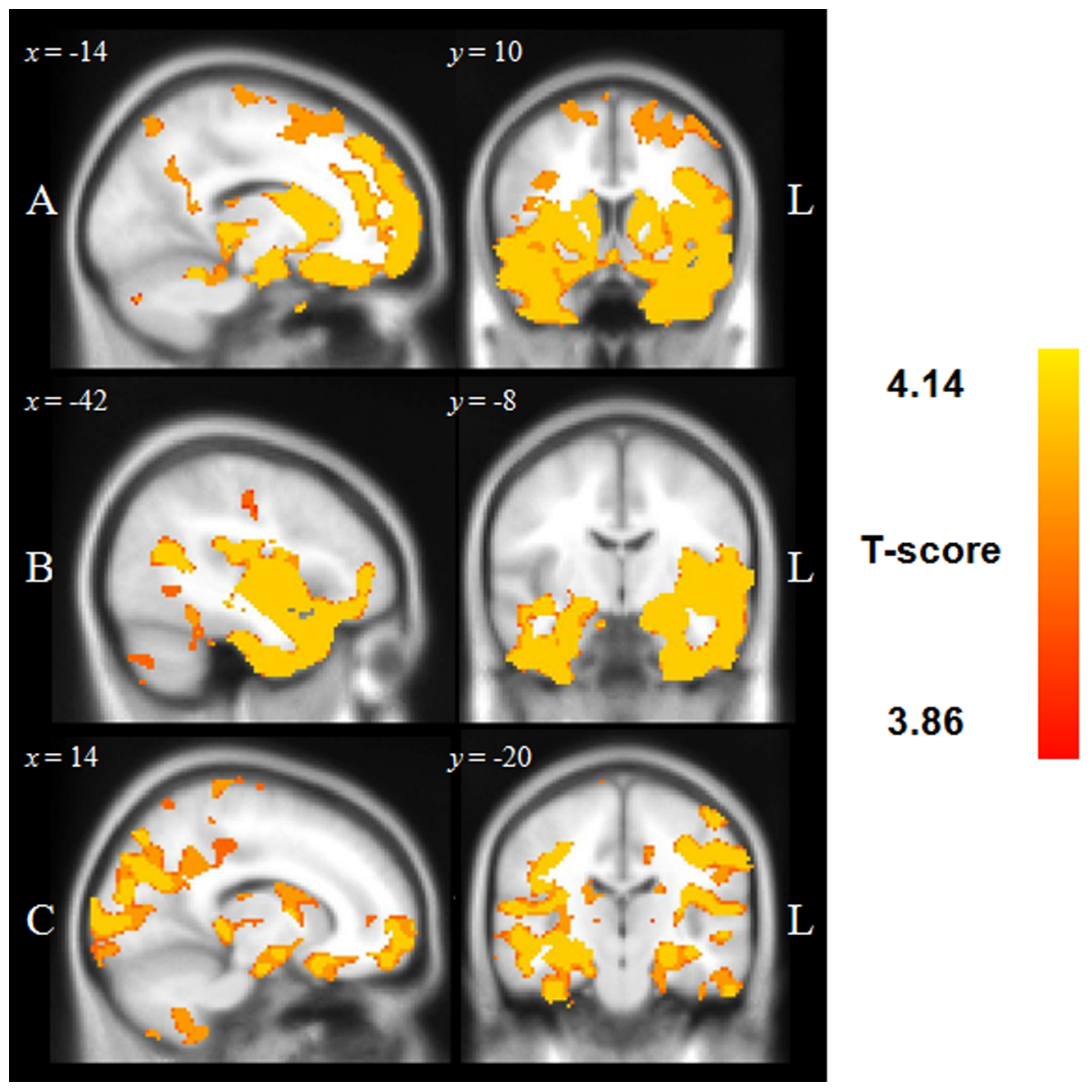
Regions of significant grey matter intensity decrease in patient groups versus Controls. (A) BvFTD (MNI coordinates: *x* = −14, *y* = 10, *z* = −22), (B) SD (*x* = −42, *y* = −8, *z* = −38), and (C) AD (*x* = 14, *y* = −20, *z* = −24). Coloured voxels show regions that were significant in the voxel-based morphometry analyses at *p*<.001 corrected for Family-Wise Error using the threshold free cluster enhancement method (tfce). Clusters are overlaid on the Montreal Neurological Institute standard brain. Age is included as a covariate in the analyses.

Figure 2 displays the patterns of grey matter density displayed by each patient group relative to Controls using the threshold free cluster enhancement method (tfce) and corrected for Family-Wise Error (FWE) at *p*<.001. All results reported indicate a decrease in grey matter intensity in patient groups relative to Controls. Briefly, bvFTD patients showed pronounced changes in the medial PFC, frontal poles, orbitofrontal, and insular cortices bilaterally, extending into the left anterior cingulate cortex and left superior frontal gyrus. Medial temporal regions of the brain were also affected including the bilateral inferior and middle temporal gyri, temporal poles, parahippocampal cortices, hippocampi, and amygdalae. Further grey matter density reduction was evident in the cerebellum bilaterally.

The SD group demonstrated characteristic grey matter intensity decrease in bilateral anterior temporal regions, extending into the temporal poles, amygdalae, hippocampi, insular and orbitofrontal cortices bilaterally, and including the left frontal pole.

Finally, AD patients showed widespread grey matter intensity decrease relative to Controls, most pronounced on the left hand side, involving medial temporal regions including the amygdala, and hippocampus bilaterally, the bilateral lateral temporal cortices, and extending to frontal regions, including the orbitofrontal cortex and frontal poles bilaterally. Posterior regions of the brain were also found to harbour significant grey matter density reduction including the right angular gyrus, bilateral supramarginal gyrus, as well as the left superior parietal lobule, left precuneus, and left lateral occipital cortices. These patterns of changes are consistent with previous reports in the literature for bvFTD [69], SD [70] and AD [71] (Table 2).

**Table 2:** Voxel-based morphometry results showing regions of significant grey matter intensity decrease for contrasts across patient samples (bvFTD, SD, AD) in comparison with Controls.

				MNI coordinates			
Contrast	Regions	Side	Number of voxels	*x*	*y*	*z*	T value
BvFTD vs. Controls	Right cerebellum, right inferior temporal gyrus, right temporal pole, right parahippocampal gyrus, right hippocampus, right amygdala, right thalamus, right insular cortex, right orbitofrontal cortex, right medial PFC, right frontal pole, extending into left frontal pole, left medial PFC, left orbitofrontal cortex, left anterior cingulate, left insular cortex, left parahippocampal cortex, left amygdala, left hippocampus, left temporal pole, left inferior temporal gyrus	B	67,718	18	−62	−64	4.11
	Cerebellum	L	2,707	−26	−84	−52	4.11
SD vs. Controls	Temporal fusiform cortex, temporal pole, parahippocampal gyrus, amygdala, hippocampus, insular cortex, orbitofrontal cortex, frontal pole	L	20,159	−24	−6	−52	4.14
	Temporal fusiform cortex, temporal pole, parahippocampal gyrus, amygdala, hippocampus, insular cortex, orbitofrontal cortex	R	6,885	34	−8	−52	4.14
	Cerebellum	L	1,250	−26	−82	−54	3.86
AD vs. Controls	Right temporal fusiform cortex, right parahippocampal gyrus, right amygdala, right hippocampus, right supramarginal gyrus, right orbitofrontal cortex, right frontal pole, extending into left frontal pole, left inferior temporal gyrus, left parahippocampal gyrus, left hippocampus, left supramarginal gyrus, left superior parietal lobule, left lateral occipital cortex, left precuneus	B	68,010	30	−2	−54	4.02

All clusters reported using threshold free cluster enhancement technique (tfce) and corrected for Family-Wise Error (FWE) at *p*<.001. For brevity only those clusters above 1,000 contiguous voxels are reported here. BvFTD = behavioural-variant frontotemporal dementia; SD = semantic dementia; AD = Alzheimer's disease; L = Left; R = Right; B = Bilateral; MNI = Montreal Neurological Institute.

An overlap analysis identified regions of grey matter intensity decrease common to all patient groups, including bilateral lateral and medial temporal regions, notably the temporal poles, amygdalae, hippocampi, as well as the bilateral insular and orbitofrontal cortices. The left superior temporal gyrus, left angular gyrus, left lateral occipital cortex, and left postcentral and precentral gyrus were also significantly affected across all of the patient groups (Table S1).

#### Grey matter correlates of autobiographical memory performance

Data from all participants (n = 50) were combined into a single general linear model to examine the neural correlates of recent versus remote ABM retrieval, irrespective of group membership, using a voxel-wise approach, with *p*<.001 uncorrected and a cluster extent threshold of 300 contiguous voxels.

Table 3 displays the neural correlates of successful internal ABM retrieval for remote and recent time periods, indicating a positive association between regions of grey matter intensity and memory performance. Retrieval of remote period internal details was found to correlate positively with grey matter intensity in the following regions bilaterally: temporal pole, orbitofrontal cortex, hippocampus, thalamus, and occipital pole. Further regions implicated in successful remote period retrieval included the left frontal pole and left medial prefrontal cortex, and the left postcentral gyrus (Table 3).

**Table 3:** Voxel-based morphometry results showing regions of grey matter intensity that covary with remote and recent autobiographical memory (ABM) retrieval in all participants combined (n = 50).

				MNI coordinates		
Contrast	Regions	Side	Number of voxels	*x*	*y*	*z*
**Remote**	Temporal pole, orbitofrontal cortex	L	1476	−18	4	−50
	Frontal pole, medial PFC	L	1408	−12	50	−28
	Parahippocampal gyrus, hippocampus (posterior), amygdala	L	1082	−14	0	−22
	Hippocampus (posterior), thalamus	R	1066	34	−20	−10
	Postcentral gyrus	L	745	−44	−12	28
	Occipital pole	L	502	−8	−102	−10
	Temporal pole, orbitofrontal cortex	R	378	38	20	−28
	Caudate, thalamus	L	331	−12	4	4
	Occipital fusiform gyrus	R	300	26	−86	−8
**Recent**	Medial PFC, frontal poles	B	1384	−2	42	−30
	Orbitofrontal cortex, caudate	L	825	−24	24	−8
	Temporal occipital fusiform cortex, hippocampus (posterior)	R	793	34	−40	−14
	Lateral occipital cortex	L	566	−34	−66	16
	Superior frontal gyrus	R	413	22	12	42
	Posterior cingulate cortex	L	397	−4	−30	26
	Inferior temporal gyrus	L	364	−62	−6	−38
	Inferior temporal gyrus	R	313	60	−30	−30
	Occipital pole	L	310	−26	−100	−8

All clusters reported using voxel-wise contrasts and uncorrected at *p*<.001. Age is included as a covariate in all contrasts. All clusters reported at *t*>3.80 with a cluster threshold of 300 contiguous voxels. L = Left; R = Right; B = Bilateral; MNI = Montreal Neurological Institute.

In contrast, recall of recent internal details was positively associated with the integrity of the medial PFC, the frontal pole, and the inferior temporal gyrus bilaterally. Further regions implicated in recent retrieval included the right hippocampus, the left posterior cingulate cortex, left lateral occipital cortices, and the left caudate (Table 3).

**Figure 3:**
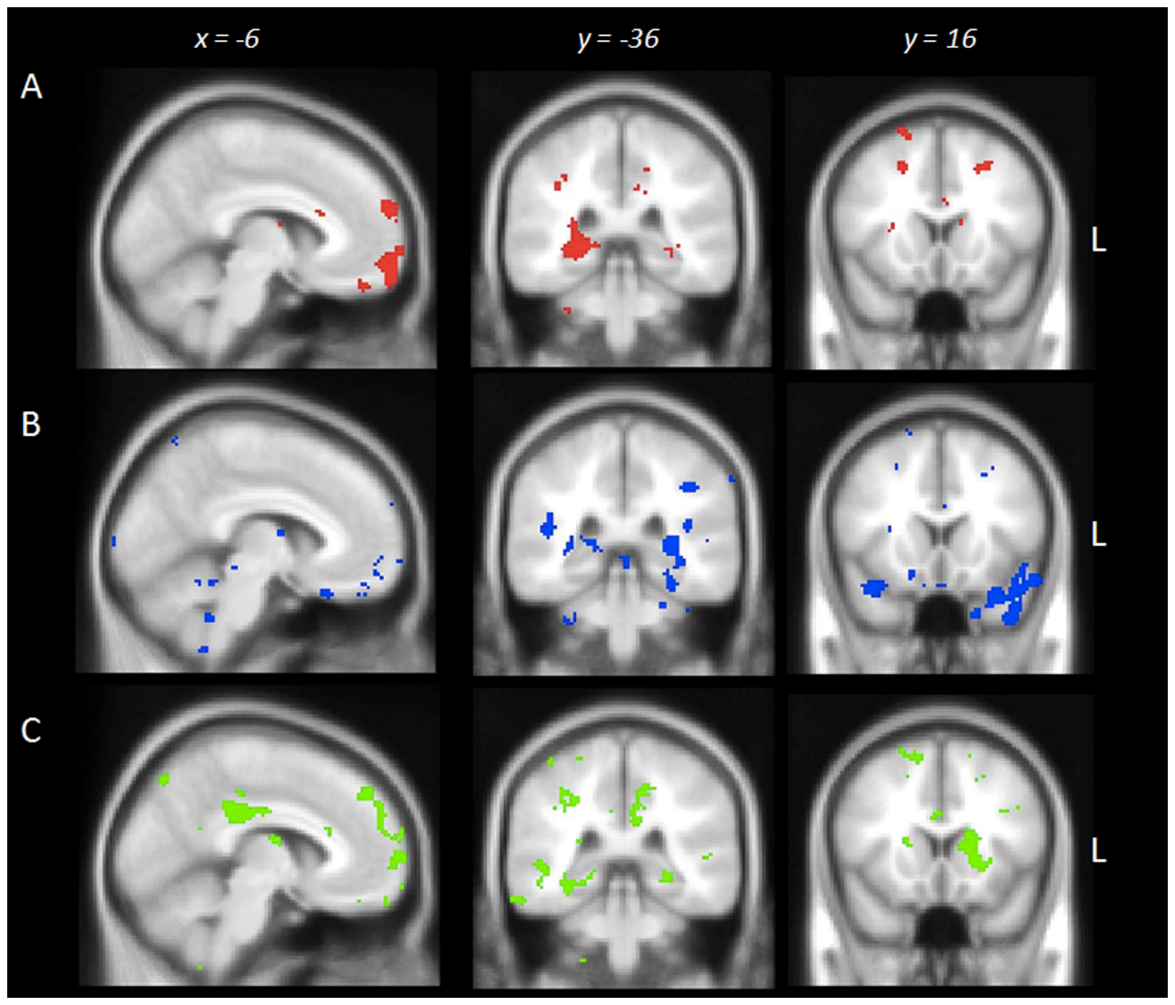
Overlap and exclusive masking results showing brain regions in which grey matter intensity correlates significantly with autobiographical memory retrieval. (A) Overlap in regions irrespective of time period; (B) Regions exclusively implicated in remote memory; (C) Regions exclusively implicated in recent memory. Coloured voxels show regions that were significant in the voxel-based morphometry covariate analyses with *p*<.001 uncorrected with a cluster threshold of 300 contiguous voxels. All clusters reported *t*>3.80 and depict a positive association between grey matter integrity and memory performance. Clusters are overlaid on the Montreal Neurological Institute standard brain. Age is included as a covariate in the analyses.

To identify the regions significantly associated with retrieval of both remote and recent ABM, we conducted an overlap analysis (Table 4). This analysis revealed the regions commonly implicated irrespective of epoch, namely the left medial PFC, left frontal pole, and the right posterior hippocampus (Figure 3A). Exclusive masking was used to identify the regions that uniquely contributed to ABM retrieval in each time period (Table 4). Integrity of bilateral temporal cortices including the temporal poles, left medial temporal structures including the left hippocampus and amygdala, and the left frontal pole correlated exclusively with remote ABM retrieval (Figure 3B). In contrast, recent ABM retrieval was positively associated with integrity of left frontal cortices including the orbitofrontal cortex and frontal pole, left lateral occipital and posterior parietal regions including the left posterior cingulate cortex and precuneus, bilateral posterior inferior temporal gyrus, right superior frontal gyrus, and the right posterior hippocampus (Figure 3C).

**Table 4:** Voxel-based morphometry results showing regions of significant grey matter intensity that correlate with ABM retrieval irrespective of time period, and regions that correlate exclusively with Remote and Recent time periods in all participants combined (n = 50).

				MNI coordinates		
Contrast	Regions	Side	Number of voxels	*x*	*y*	*z*
Overlap	Medial PFC, frontal pole	L	636	−6	46	−26
	Hippocampus (posterior)	R	472	40	−22	−12
Remote	Temporal pole, inferior temporal gyrus (anterior)	L	1290	−16	2	−46
	Hippocampus, amygdala, putamen	L	947	−14	−14	−24
	Postcentral gyrus, precentral gyrus	L	661	−46	−14	28
	Frontal pole	L	588	−14	44	−28
	Temporal pole, middle temporal gyrus	R	378	48	6	−28
	Superior temporal gyrus, supramarginal gyrus	R	376	44	−32	4
Recent	Orbitofrontal cortex, caudate, putamen	L	540	−26	22	−10
	Lateral occipital cortex, precuneus	L	507	−36	−64	14
	Frontal pole	L	396	−14	58	−16
	Posterior cingulate cortex	L	346	−6	−36	26
	Superior frontal gyrus	R	331	20	20	44
	Inferior temporal gyrus (posterior)	L	331	−62	−6	−40
	Inferior temporal gyrus (posterior)	R	309	60	−30	−30
	Hippocampus (posterior), lingual gyrus	R	306	34	−36	−16

All results reported using voxel-wise contrasts and uncorrected at *p*<.001. Age is included as a covariate in all contrasts. All clusters reported at *t*>3.80 with a cluster threshold of 300 contiguous voxels. L = Left; R = Right; MNI = Montreal Neurological Institute.

### Diffusion tensor imaging

#### Group differences in fractional anisotropy

Table 5 displays the patterns of fractional anisotropy decrease for each of the patient groups relative to Controls. Diffusion tensor imaging data were not available for 1 bvFTD patient. Analysis of whole-brain fractional anisotropy across groups, controlling for age, revealed the following patterns. Compared to Controls, bvFTD patients showed reduced fractional anisotropy bilaterally in the inferior frontooccipital, uncinate, inferior and superior longitudinal fasciculi, as well as the anterior thalamic radiation, forceps minor, forceps major, and cingulum (cingulate gyrus and hippocampal parts). SD patients, by contrast, showed reduced fractional anisotropy predominantly in the left inferior longitudinal fasciculus, and left uncinate fasciculus. Finally AD patients displayed bilateral reduced fractional anisotropy involving the forceps major, forceps minor, the inferior frontooccipital, inferior longitudinal, and uncinate fasciculi, as well as the cingulum (cingulate gyrus) (Table 5). These patterns of fractional anisotropy changes are in keeping with previous reports in the literature for bvFTD and SD [72] and AD [73] patients.

**Table 5:** Tract-based spatial statistics results showing regions of white matter integrity decrease in each of the patient groups compared with Controls^a^

				MNI coordinates			
Contrast	Tracts	Side	Number of voxels	*x*	*y*	*z*	T value
**BvFTD versus Controls**	Inferior longitudinal fasciculus, uncinate fasciculus, inferior fronto-occipital fasciculus, superior longitudinal fasciculus, anterior thalamic radiation, forceps minor, forceps major, cingulum (cingulate gyrus), cingulum (hippocampal part), superior longitudinal fasciculus (temporal part)	B	64,986	−28	0	−36	4.14
**SD versus Controls**	Inferior longitudinal fasciculus, uncinate fasciculus	L	2,096	−34	−3	−34	2.53
**AD versus Controls**	Forceps major, forceps minor, inferior fronto-occipital fasciculus, inferior longitudinal fasciculus, uncinate fasciculus, cingulum (cingulate gyrus)	B	31,691	−24	−56	11	3.49

a Diffusion tensor imaging data not available for 1 bvFTD patient. Age is included as a covariate in all contrasts. All clusters reported using threshold-free cluster enhancement method and corrected for Family-Wise Error (FWE) at *p*<.05. BvFTD = behavioural-variant frontotemporal dementia; SD = semantic dementia; AD = Alzheimer's disease; L = Left; B = Bilateral; MNI = Montreal Neurological Institute.

#### Fractional anisotropy correlations with autobiographical memory performance

Fractional anisotropy values of white matter tracts connecting grey matter regions found to correlate positively with ABM retrieval in the voxel-based morphometry analyses were investigated. Masks for the following white matter tracts, taken from the Johns Hopkins probabilistic white matter atlas [74] were created; uncinate fasciculus (connecting medial prefrontal and anterior temporal brain regions), cingulum (running along the surface of the corpus callosum and hippocampal parts), inferior longitudinal fasciculus (connecting the temporal lobes and occipital lobes), superior longitudinal fasciculus (connecting the frontal, temporal, parietal and occipital lobes), and the forceps minor (connecting medial and lateral prefrontal regions). Fractional anisotropy values for each white matter tract were extracted and exported into SPSS. Pearson R correlations, corrected for multiple comparisons at *p*<.01, were then run to investigate positive associations between fractional anisotropy values and recent and remote ABM performance.

**Figure 4:**
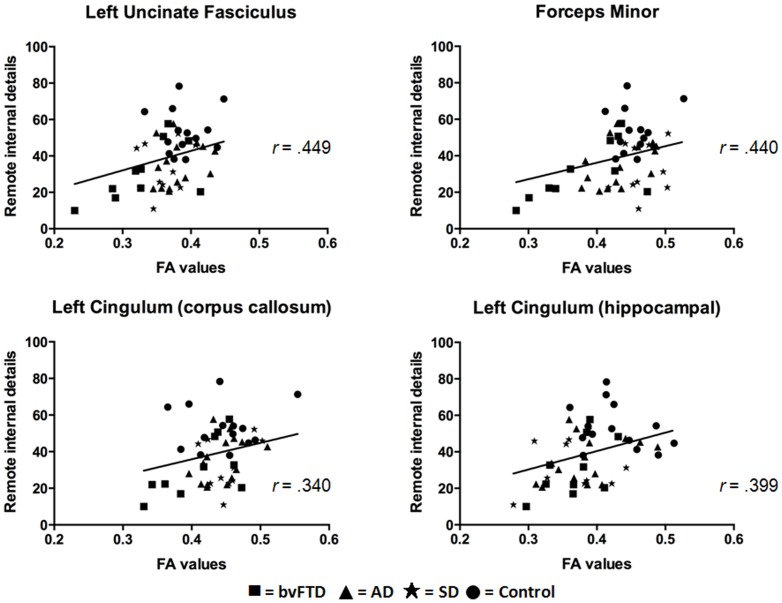
Relationship between fractional anisotropy (FA) values in white matter tracts of interest and remote memory. All participants included in the analyses (n = 49). Age is included as a covariate in the analyses. Plotted data depict a positive association between fractional anisotropy values and memory performance, with the magnitude of this relationship calculated using Pearson's R correlations (r).

**Figure 5:**
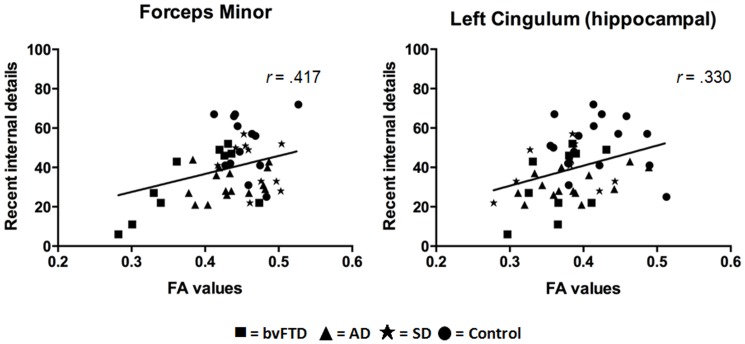
Relationship between fractional anisotropy (FA) values in white matter tracts of interest and recent memory. All participants included in the analyses (n = 49). Age is included as a covariate in the analyses. Plotted data depict a positive association between fractional anisotropy values and memory performance, with the magnitude of this relationship calculated using Pearson's R correlations (r).

Fractional anisotropy values in the left uncinate fasciculus (*r* = .449, *p* = .001), left cingulum (corpus callosum part: *r* = .340, *p* = .008; hippocampal part: *r* = .399, *p* = .002), and the forceps minor (*r* = .440, *p* = .001) were found to correlate significantly with remote ABM retrieval (Figure 4). In contrast, only fractional anisotropy values in the forceps minor (*r* = .417, *p* = .001) and the left cingulum (hippocampal part; *r* = .330, *p* = .010) were significantly associated with recent ABM performance (Figure 5).

## Discussion

This study is the first to contrast the neural substrates of recent and remote ABM using a combination of whole-brain voxel-based morphometry analyses and tract-based spatial statistics in neurodegenerative disorders. Distinct grey matter correlates common to both recent and remote time periods were found, including the right posterior hippocampus, left medial prefrontal and frontopolar cortices. Notably, however, discrete regions potentially specialised for the retrieval of information specific to each time period were identified. Remote ABM performance was associated with integrity of the bilateral anterior temporal cortices including the temporal poles, whereas retrieval of recent ABMs was associated with the integrity of left posterior parietal regions including the posterior cingulate cortex. Associations with white matter tract integrity were also found: the forceps minor and left hippocampal portion of the cingulum bundle were significantly associated with both recent and remote retrieval, while a distributed set of predominantly left-lateralised white matter tracts were implicated for remote retrieval. Our findings point towards commonalities and differences between the grey and white matter regions implicated in remote and recent recall and shed further light on the neurocognitive mechanisms supporting ABM retrieval.

Using neurodegenerative disorders as lesion models for declarative memory processes, we have confirmed that ABM retrieval depends upon the integrity of a distributed neural network. Notably, this network comprises not only medial temporal, but also lateral temporal, medial prefrontal and frontopolar structures, as well as posterior regions in the parietal and occipital lobes. Our findings converge with a large body of literature from functional neuroimaging studies in healthy individuals revealing a core memory network which subtends successful retrieval of events from the past [9], [11]. Importantly, our study points to the hippocampus, medial prefrontal, and frontopolar cortices, as pivotal structures for successful ABM retrieval irrespective of time period [19]–[21], [75].

The temporal duration of hippocampal involvement in the retrieval of ABMs from across the lifespan represents a heavily debated issue within the literature. Here, we demonstrate hippocampal involvement in ABM retrieval regardless of the age of the memory, with contributions evident for recent events (i.e., less than 1 year old), as well as for considerably older episodes, stretching back over 40 years prior. Notably, our diffusion tensor imaging analyses reveal that integrity of the hippocampal portion of the left cingulum bundle, projecting from the posterior cingulate gyrus to the entorhinal cortex, also correlates with ABM retrieval irrespective of time period, providing converging grey and white matter evidence in support of a time-invariant role of medial temporal regions for ABM retrieval. These findings resonate with the multiple trace theory [13], [14] which delineates a permanent role for the hippocampus in supporting the rich recollection of previously experienced events. Subregions of the hippocampus have been differentially implicated across ABM studies with recent memories shown to activate the anterior hippocampus [19], whereas remote memories appear to be represented more evenly across the hippocampal formation [15]. Recently, both recent and remote memories have been shown to be represented within the anterior and posterior hippocampus, however, the posterior hippocampus appears more sensitive to the retrieval of remote memories [16]. In this study, we found evidence only for posterior hippocampal involvement in ABM, however, this finding may in part reflect methodological differences in the sampling of ABMs and demarcation of recent versus remote time periods, as well as the use of a post hoc correlation approach in neurodegenerative disorders. Finally, some hippocampal lateralisation effects were observed, with the right hippocampus implicated irrespective of time period, whereas the left hippocampus correlated exclusively with remote memory retrieval. Interestingly, however, only the left hippocampal portion of the cingulum bundle was found to correlate with ABM retrieval irrespective of epoch. Considerable variation exists within the literature regarding the response of the hippocampii according to the age of the retrieved memory, with some studies reporting bilateral hippocampal recruitment irrespective of time period [21], whereas others have demonstrated a time invariant role for the left hippocampus and increasing activation of the right hippocampus according to the recency of the memory [76]. While our overall findings mesh well with existing reports in the literature emphasising a permanent role for the hippocampus in ABM retrieval, our divergent lateralisation effects caution against a simple mapping of results from lesion studies to functional activation studies conducted in healthy individuals. We suggest that functional activation studies in the disease groups in question will serve to considerably strengthen our understanding of the specific neural regions implicated in ABM dysfunction across the lifespan, as has been conducted in semantic dementia [36].

Despite appreciable overlap in the neural substrates of recent and remote ABM retrieval, we further identified discrete neural correlates for each epoch. For remote retrieval, in which memories stretching back over 40 years were sampled, the largest grey matter cluster to correlate exclusively with remote memory resided in the left temporal pole and left anterior inferior temporal gyrus, regions specialised for semantic memory [5], [77]. This finding is consistent with the proposal that old memories undergo a process of semanticisation [78]. Over time, repeated recollection and rehearsal of remote memories facilitates abstraction of the gist of the episode, divested of its accompanying sensory-perceptual details [25]. This process of abstraction therefore produces a largely schematic or overgeneral account of the formerly evocative event [4], [7]. The sizeable contribution of semantic processing regions to remote memory retrieval found here suggests that, over time, episodic ABMs lose much of their autonoetic flavour, leading to a reliance on predominantly semantic or gist-based representations which represent the most accessible and efficient route of access [79]. Our tract-based spatial statistics results revealed that fractional anisotropy values from predominantly left-lateralised white matter tracts connecting frontal, temporal, and occipital regions (uncinate fasciculus, cingulum, forceps minor) were significantly related to remote ABM retrieval, suggesting the importance of connectivity between a distributed network of regions in supporting recollection of events from the distant past [9].

For retrieval of recent memories (i.e., events that had occurred within the previous year), our voxel-based morphometry analyses revealed the involvement of the right posterior hippocampus, left orbitofrontal, left occipital and bilateral posterior inferior temporal cortices. Most striking, however, was the emergence of the left posterior cingulate cortex as a crucial structure for recent retrieval. Our finding of posterior cingulate cortex involvement exclusively in the recent period is in keeping with prior reports of its preferential activation in healthy individuals for recent ABMs [8], [19]. This midline posterior structure represents a site of particular interest as its functional specialisation in the context of ABM retrieval remains poorly understood. In contrast with the widespread involvement of multiple white matter tracts for remote retrieval, our diffusion tensor imaging analyses revealed that only the forceps minor and the left cingulum bundle (hippocampal portion) were significantly related to recent ABM retrieval. Again, these findings converge to suggest the involvement of frontal and posterior hippocampal/cingulate regions in the successful recollection of recent events. The posterior cingulate has been proposed to facilitate rich contextual re-experiencing [15], and the generation of visuospatial imagery [80], with further studies pointing to its role in the coding of personally familiar places [81] and self-referential processing [82]. With increasing recency, it is likely that the ABMs retrieved by participants are imbued with a sense of recollection, vivid visual imagery, and self-referential connotations [83]. Indeed, recency of events has previously been associated with increased instances of rich autonoetic re-experiencing [3], [83]. Accordingly, the preferential involvement of the posterior cingulate cortex for recent period retrieval suggests that this region may be particularly well suited to support the recollective endeavour.

A number of methodological issues warrant consideration in the current context. Our grey matter voxel-based morphometry covariate results did not survive conservative correction for multiple comparisons (i.e., Family-Wise Error) and were therefore reported uncorrected at *p*<.001. To guard against the potential for false positive results, however, we applied strict cluster extent thresholds of 300 contiguous voxels in the covariate analyses. Given our sample size, the application of stringent cluster extent thresholds, and our *a priori* hypotheses, we are confident that our results do not represent false positive findings. The use of dementia syndromes as lesion models for ABM processes presents a number of challenges, one of which concerns the possibility of confabulation in the bvFTD patient group, particularly with the use of structured probing in the high retrieval condition. While this could not be definitively ruled out, details were cross-checked with carers where the veracity of memories was in doubt. It is also important to note that some of the regions to emerge in our voxel-based morphometry analyses for ABM processes have been implicated in behavioural disruption in neurodegenerative disorders [84]. In general, however, the neural correlates of ABM dysfunction identified here correspond remarkably well with the key regions of the core ABM network expounded in healthy individuals [9].

The classification of what constitutes a “recent” memory varies considerably in the literature, ranging from relatively extended periods of time (e.g., within the past 5 years) to more discrete snapshots (e.g., within the past month). It remains unclear whether the differential dissection of time periods impacts the neural correlates of ABM retrieval and this represents an interesting avenue for future investigation. A further caveat concerns the differential statistical thresholds used in the grey and white matter neuroimaging analyses. Future studies with larger sample sizes will permit the use of one statistical threshold across all levels of analyses. One further issue of relevance to the hippocampal contributions to ABM recall concerns the recollective quality of the events retrieved. Previous studies have pointed to the modulating effect of specific recollective aspects of retrieval, with comparable patterns of activation reported for recent and remote ABMs that are matched in terms of vividness [20], [85]. As we did not collect subjective ratings from our participants, it is not possible to determine how recollective factors, such as vividness, visual imagery, and emotional tone, potentially modulate the specific neural correlates of recent and remote ABM retrieval reported here. We suggest that future investigations incorporating subjective experiential ratings will be important to clarify the functional specialisation of specific brain regions for recent and remote memory retrieval. Finally, we did not have access to functional connectivity data in our participant cohort, however, it will be crucial to incorporate such measures in future studies to elucidate how alterations in functional connectivity between specific regions disrupt the capacity for ABM retrieval in neurodegenerative disorders.

## Conclusions

Using neurodegenerative disorders as lesion models for declarative memory processes, we have demonstrated that recent and remote ABM retrieval is associated with the integrity of common and unique grey and white matter structures. Our findings suggest that regions specialised for semantic memory play an important role in the retrieval of distant memories, whereas midline posterior parietal structures may be preferentially involved for more recent events. Irrespective of time period, however, ABM retrieval appears to be significantly associated with the integrity of the hippocampus, resonating with current theories emphasising a time-invariant role for the medial temporal lobes in retrieving events from the past.

## Supporting Information

Table S1 **Voxel-based morphometry results showing regions of grey matter intensity decrease common to all patient groups (AD, bvFTD, SD) relative to Controls.**
(DOCX)
