# Supplementary material for: Grey and White Matter Correlates of Recent and Remote Autobiographical Memory Retrieval – Insights from the Dementias
Source: PLoS One. 2014 Nov 14;9(11):e113081. doi: 10.1371/journal.pone.0113081 (PMC4232597; doi:10.1371/journal.pone.0113081)
Supplement: Table S1 — Voxel-based morphometry results showing regions of grey matter intensity decrease common to all patient groups (AD, bvFTD, SD) relative to Controls. (DOCX) [file pone.0113081.s001.docx]

**Table S1.** Voxel-based morphometry results showing regions of grey matter intensity decrease common to all patient groups (AD, bvFTD, SD) relative to Controls.

| Contrast | Regions | Side | Number of voxels | MNI  coordinates | | |
| --- | --- | --- | --- | --- | --- | --- |
|  |  |  |  | *x* | *y* | *z* |
| Regions of overlap | Temporal fusiform cortex, temporal pole, inferior temporal gyrus, parahippocampal cortex, hippocampus, amygdala, insular cortex, orbitofrontal cortex, frontal pole, inferior frontal gyrus. | L | 10,146 | -38 | -4 | -50 |
|  | Temporal fusiform cortex, temporal pole, superior temporal gyrus, insular cortex, orbitofrontal cortex, amygdala, hippocampus. | R | 4,512 | 34 | -8 | -52 |
|  | Inferior temporal gyrus, angular gyrus, lateral occipital cortex. | L | 600 | -46 | -56 | -8 |
|  | Cerebellum | L | 385 | -22 | -82 | -54 |
|  | Postcentral gyrus, precentral gyrus | L | 228 | -42 | -14 | 32 |
|  |  |  |  |  |  |  |

All results presented using the threshold free cluster enhancement method and corrected for Family-Wise Error at *p* < .001. All clusters reported at *t* > 3.50 with a cluster threshold of 100 contiguous voxels. L = Left; R = Right; MNI = Montreal Neurological Institute.
